# (Dimethyl sulfoxide-κ*O*)[3-hydr­oxy-2-hydroxy­methyl-2-(3-meth­oxy-2-oxido­benzyl­ideneamino-κ^2^ *O* ^2^,*N*)propanolato-κ*O*]dioxomolybdenum(VI). Corrigendum

**DOI:** 10.1107/S1600536810007476

**Published:** 2010-04-14

**Authors:** Yan Sui, Xiao-Niu Fang, Qiu-Yan Luo, Hong-Mei Chen, Meng-Qiang Zhou

**Affiliations:** a JiangXi Province Key Laboratory of Coordination Chemistry, College of Chemistry & Chemical Engineering, JingGangShan University, 343009 Ji’an, JiangXi, People’s Republic of China

## Abstract

The crystal structure of the title compound in the paper by Sui, Fang, Luo, Chen & Zhou [*Acta Cryst.* (2006), E**62**, m1994–m1996] has been rerefined to allow for identification of a disordered dimethyl sulfoxide ligand.

## Abstract 2

Corrigendum to *Acta Cryst.* (2006), E**62**, m1994–m1996.

The structure reported by Sui *et al.* (2006) ▶ has been rerefined. The compound was originally determined by Rao *et al.* [*J. Chem. Soc. Dalton Trans.* (1998) ▶, 2383] and has been redetermined here to a significantly higher precision of the lattice parameters [*a* = 14.3130 (7) Å, *b* = 9.2596 (5) Å and *c* = 14.8563 (7) Å here *versus* *a* = 14.305 (3) Å, *b* = 9.249 (2) Å and *c* = 14.860 (3) Å reported by Rao *et al.*], bond lengths and s.u. values [*e.g.* Mo1—O6 = 1.6937 (17) Å here *versus* Mo1—O6 1.697 (4) Å reported by Rao *et al.*; *R* = 0.022 here *versus R* = 0.050 reported by Rao *et al.*]. The results of the current redetermination allow the identification of a disordered dimethyl sulfoxide ligand and a clarification of the nature of the intra- and inter­molecular hydrogen bonding.

## Experimental

### 

#### Crystal data

[Mo(C_12_H_15_NO_5_)O_2_(C_2_H_6_OS)]
*M* *_r_* = 459.32
Monoclinic, 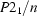

*a* = 14.3130 (7) Å
*b* = 9.2596 (5) Å
*c* = 14.8563 (7) Å
β = 115.324 (1)°
*V* = 1779.74 (15) Å^3^
*Z* = 4
Mo *K*α radiation
μ = 0.90 mm^−1^
*T* = 295 K
0.56 × 0.39 × 0.35 mm

#### Data collection

Bruker APEXII area-detector diffractometer
Absorption correction: multi-scan (*SADABS*; Bruker, 2004 ▶) *T*_min_ = 0.632, *T*_max_ = 0.746
10668 measured reflections
3278 independent reflections
3056 reflections with *I* > 2σ(*I*)
*R*_int_ = 0.024

#### Refinement

*R*[*F* ^2^ > 2σ(*F* ^2^)] = 0.022
*wR*(*F* ^2^) = 0.064
*S* = 1.07
3278 reflections
259 parameters
40 restraints
H-atom parameters constrained
Δρ_max_ = 0.36 e Å^−3^
Δρ_min_ = −0.44 e Å^−3^

### 

Data collection: *APEX2* (Bruker, 2004 ▶); cell refinement: *APEX2* (Bruker, 2004 ▶); data reduction: *APEX2* (Bruker, 2004 ▶); program(s) used to solve structure: *SHELXS97* (Sheldrick, 2008 ▶); program(s) used to refine structure: *SHELXL97* (Sheldrick, 2008 ▶); molecular graphics: *SHELXL97* (Sheldrick, 2008 ▶); software used to prepare material for publication: *SHELXL97* (Sheldrick, 2008 ▶).

## Supplementary Material

Crystal structure: contains datablocks global, I. DOI: 10.1107/S1600536810007476/ng9064sup1.cif

Structure factors: contains datablocks I. DOI: 10.1107/S1600536810007476/ng9064Isup2.hkl

## Figures and Tables

**Table 1:** Hydrogen-bond geometry (Å, °)

*D*—H⋯*A*	*D*—H	H⋯*A*	*D*⋯*A*	*D*—H⋯*A*
C1—H1⋯O5	0.93	2.22	2.874 (3)	127
C14—H14*E*⋯O7^i^	0.96	2.45	3.343 (4)	154
C8—H8*A*⋯O6^ii^	0.96	2.54	3.475 (4)	164
C11—H11*A*⋯O7^iii^	0.97	2.57	3.534 (3)	170
O5—H5*A*⋯O1^iii^	0.82	2.02	2.834 (2)	175
O5—H5*A*⋯O2^iii^	0.82	2.53	2.970 (2)	115
O4—H4⋯O3^iv^	0.82	1.99	2.801 (2)	171

Symmetry codes: (i) 
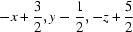
; (ii) 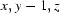; (iii) 
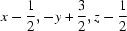
; (iv) 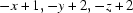.
